# Utilization of Sewage Sludge-Derived Biochar as a Functional UV Stabilizer in Recycled Poly(ethylene terephthalate) Nanocomposite Materials

**DOI:** 10.3390/polym18141758

**Published:** 2026-07-19

**Authors:** Nikolaos Pardalis, Lazaros Karagiannidis, Panagiotis A. Klonos, Eleftheria Maria Pechlivani, Rafail O. Ioannidis, Apostolos Kyritsis, Konstantinos Chrisafis, Dimitrios N. Bikiaris

**Affiliations:** 1 Laboratory of Polymer Chemistry and Technology, Department of Chemistry, Aristotle University of Thessaloniki, GR54124 Thessaloniki, Greece; npardal@chem.auth.gr (N.P.); rafailio@chem.auth.gr (R.O.I.); dbic@chem.auth.gr (D.N.B.); 2 Laboratory of Advanced Materials and Devices, Department of Physics, Aristotle University of Thessaloniki, GR54124 Thessaloniki, Greece; lkaragi@physics.auth.gr (L.K.); hrisafis@physics.auth.gr (K.C.); 3 Dielectrics Research Group, Department of Physics, National Technical University of Athens, GR15780 Athens, Greece; pklonos@central.ntua.gr (P.A.K.); akyrits@central.ntua.gr (A.K.); 4 Centre for Research and Technology Hellas, Information Technologies Institute, 6th km Charilaou-Thermi Road, GR57001 Thessaloniki, Greece

**Keywords:** recycled PET, sewage sludge-derived biochar, nanocomposites, photo-oxidative degradation, UV stabilization, thermal degradation, circular economy

## Abstract

This work investigates the incorporation of sewage sludge-derived biochar (BC) into a recycled poly(ethylene terephthalate) (rPET) matrix at loadings ranging from 0.5 to 5% wt. in order to develop sustainable nanocomposite materials. The materials were comprehensively characterized using complementary structural, thermal, and morphological techniques to evaluate the effect of BC on the properties of the recycled polymer. XRD, DSC, and PLM analyses demonstrated that BC acts as a heterogeneous nucleating agent, without significantly altering the final crystalline fraction of rPET, while SEM observations confirmed the homogeneous dispersion of particles within the matrix. TGA and Py–GC/MS further indicated that BC moderates thermal degradation, reducing the relative formation of low-molecular-weight degradation products and promoting the retention of larger terephthalate-containing fragments. Accelerated UV irradiation experiments further demonstrated the protective role of BC against photo-induced degradation. Intrinsic viscosity (IV) measurements showed that BC-containing nanocomposites exhibited a smaller molecular-weight reduction after UV exposure compared to neat rPET, indicating reduced chain scission, while complementary DSC analyses confirmed improved preservation of thermal transitions after aging. Overall, sewage sludge-derived biochar is demonstrated to be a promising multifunctional additive for rPET, acting both as a nucleating agent and a UV stabilizer. This approach provides an alternative route for the valorization of two waste streams, contributing to enhanced materials in a circular economy framework.

## 1. Introduction

The increasing amount of plastic waste has emerged as a major environmental challenge, driving remarkable efforts towards the development of sustainable materials and effective recycling methods. Among the most used polymers, poly(ethylene terephthalate) (PET) is extensively applied in packaging, textiles, and engineering applications thanks to its excellent mechanical and thermal performance. However, the large amount of post-consumer PET waste has generated concern, necessitating the efficient utilization of recycled resources [1]. Despite its industrial relevance, recycled PET (rPET) usually displays weaker properties compared to its virgin counterpart, mainly caused by degradation effects taking place during recycling procedures and service life [2,3,4]. Particularly, repeated thermal and mechanical stresses can cause chain scission, thus resulting in reduction in the molecular weight and deterioration of the final properties [5]. Consequently, strategies aimed at improving the performance, durability, and resistance of rPET to thermal and environmental degradation has become an area of considerable interest.

To mitigate these problems, various modification approaches have been explored in order to enhance its performance. A main problem in rPET-based materials has to do with the reduction in molecular weight due to thermo-mechanical processing [4,6]. This is known to directly affect mechanical properties and thermal stability. With regard to this problem, the incorporation of suitable fillers has been considered as a straightforward route to improve stiffness, thermal resistance, and crystallization kinetics [7,8,9,10,11], while reactive additives can promote chain extension and, in some cases, the recovery of the molecular weight during melt processing. Among these, multifunctional chain extenders such as pyromellitic dianhydride (PMDA) have been reported to interact with the end groups of PET, restoring the molecular weight during melt processing [12]. However, the balance in properties remains complex, as the extensive amount of additives may cause negative effects, such as chain mobility restriction and limited crystallization. Therefore, the selection of the appropriate filler, as well as the right amount, is critical for high-performance materials [13,14].

In recent years, biochar has gained significant interest in the polymer composite field [15]. It is a carbon-rich material that can be obtained through the thermochemical conversion of biomass under limited oxygen conditions and can be derived from a variety of waste resources, including agricultural residues and sewage sludge [16,17,18]. In particular, sewage sludge is an abundant waste from wastewater streams, and its conversion into biochar offers a carbon-rich material, perfect for composite applications [19,20,21,22]. The utilization of such waste streams as polymer fillers fully agrees with the concept of waste valorization, contributing to the development of environmentally friendly and cost-effective materials [23,24,25,26]. Owing to its porous structure, high carbon content, and functional groups, biochar has the potential to positively impact both the physical and the structural properties of the polymer matrix. Literature has mentioned that it can act as a reinforcing filler and, in some cases, as a nucleating agent for semicrystalline polymers. Nevertheless, its effectiveness depends on a number of parameters, including its origin, pyrolysis conditions, concentration, and particle diameter within the polymer matrix [27], making it worthy of further investigation.

In addition to their reinforcing characteristics, carbon-based fillers have been reported to mitigate environmental degradation as their light-absorbing properties can lower the penetration of ultraviolet (UV) radiation into the polymer matrix [28]. PET is known to be susceptible to photo-oxidative degradation, which promotes chain scission, molecular weight reduction, and deterioration of mechanical performance [29]. Prolonged environmental exposure may further induce embrittlement and fragmentation of the PET polymeric backbone, contributing to the formation of secondary microplastics (MPs). Recent studies have identified UV irradiation as one of the major drivers of environmental weathering of PET and other commodity polymers, accelerating the formation of MP particles through photo-induced degradation processes [30,31,32]. From this perspective, biochar can potentially act as an effective UV stabilizer, limiting degradation processes and extending the service lifetime of PET-based materials under UV irradiation [33]. This aspect is an additional advantage of biochar that has not been extensively studied in recycled PET systems. To date, limited information is available regarding the combined influence of sewage sludge-derived biochar on the crystallization behavior, thermal degradation pathways, and UV-aging resistance of recycled polymers.

In this study, rPET/BC nanocomposites were produced via melt mixing, incorporating sewage sludge-derived biochar, addressing the relatively limited literature on these materials. The aim was to observe the effect of biochar ratio on the structural, thermal, morphological, and dielectric properties of the nanocomposites. In addition, the potential contribution of biochar to the UV stability of the nanocomposites was also evaluated using intrinsic viscosity (IV) measurements and complementary DSC. The results provide insight into the role of biochar as a multifunctional additive in recycled polymer systems. Particular emphasis is given to the influence of biochar on the thermal and photo-oxidative degradation behavior of rPET, assessing its ability to reduce degradation-induced molecular weight loss. Such an approach may contribute not only to the valorization of two waste streams but also to the development of more durable recycled plastics with improved resistance to environmental aging.

## 2. Materials and Methods

### 2.1. Materials

#### 2.1.1. rPET and Biochar

Recycled polyethylene terephthalate (rPET) was obtained in the form of pellets by DION ABETE (Thessaloniki, Greece). Transparent post-consumer PET bottles with IV 0.49 dL/g were shredded, separated from caps and labels, subjected to hot water and NaOH, dried, and subsequently pelletized using an extruder/pelletizer system. The biochar (BC) was produced from sewage sludge digestate originating from a municipal wastewater treatment plant operated by Lahti Aqua Ltd. in Lahti, Finland. The digestate had a moisture content of 67.0% prior to processing. Feedstock pre-treatment consisted of anaerobic digestion at Lahti Aqua followed by thermal drying at the LAB University of Applied Sciences before pyrolysis. The production was carried out at laboratory scale using a batch pyrolysis reactor with a batch size of 11.8 kg. The maximum process temperature was 650 °C. The heating program consisted of temperature increments of 50, 200, 350, 500, and 650 °C/h. The residence time at the final temperature was 2 h and the yield obtained under these conditions was 18.1% (dry mass basis). The as-received BC was ball-milled to reduce its particle size and subsequently characterized by dynamic light scattering (DLS) analysis, giving an average particle diameter of ~740 nm with a PDI of 0.13. This narrow distribution and relatively small size are considered adequate for good dispersion in the polymer matrix, thus promoting enhanced material properties. Pyromellitic dianhydride (PMDA) was obtained from Fluka, Switzerland, with a purity of 99.7%.

#### 2.1.2. Nanocomposite Materials

rPET was melt-blended with varying loadings of BC to prepare a series of nanocomposite materials. The BC loadings ranged from 0.5 to 5% wt., while the total mass of each formulation was kept constant at 50 g (Table 1). The mixtures were processed in a twin-screw extruder at 270 °C for 10 min, and pyromellitic dianhydride (PMDA) was incorporated at 0.5% wt. as a chain extender. Due to its ability to react with terminal hydroxyl and carboxyl groups of degraded PET [34,35,36], PMDA has been mentioned to increase molecular weight, while in this particular system it can also act as a compatibilizer by promoting interfacial interactions between the rPET matrix and the BC. The unfilled rPET sample served as the reference for comparative analysis.

#### 2.1.3. UV Aging Procedure

Accelerated UV aging was performed in a UV chamber with six UVC lamps at 254 nm. A thin film (~0.3 mm) of each sample was placed at a distance of 25 cm from the lamp. A constant aging cycle of 6 h of irradiation followed by 2 h without irradiation was applied for a total of 90 days. The samples were flipped every 7 days in order to ensure uniform exposure. Although UVC irradiation does not reproduce the spectral distribution of natural sunlight, it is widely employed as an accelerated aging method to rapidly induce photo-oxidative degradation and enable the comparative evaluation of the UV stability of polymeric materials under controlled and severe exposure conditions [37,38,39]. It is important to mention that UV degradation is a surface-driven phenomenon, but IV and DSC measurements reflect the entire specimen thickness. Therefore, the results should be considered for comparative purposes rather than as absolute measures of degradation at the exposed surface.

### 2.2. Characterization Techniques

#### 2.2.1. Fourier Transform Infrared Spectroscopy (FTIR)

IR measurements were performed in order to examine the chemical structure of the nanocomposites. ATR spectra were recorded on an IRTracer100 (Shimadzu, Kyoto, Japan) equipped with a QATR 10 Single-Reflection ATR Accessory with a Diamond Crystal. All spectra were collected from 4000 to 450 cm^−1^, resolution 4 cm^−1^, and 32 co-added scans. The spectra were further baseline-corrected and normalized.

#### 2.2.2. X-Ray Diffraction (XRD)

The XRD spectra were recorded at 25 °C using a MiniFlex II XRD system (Rigaku Co., Tokyo, Japan) with Cu Ka radiation (0.154 nm) over the 2θ range from 5° to 45° with a scanning rate of 1 °C/min. The % crystallinity was calculated from the XRD graphs using the equation of Hay (1) [40]:

(1) $$X_{c}\;={\left(1+\frac{A_{am}}{A_{cr}}\right)\;}^{-1}\;\times \;100\%,$$

where A_am_ is the area of the amorphous halo and A_cr_ is the area of the crystalline peaks.

#### 2.2.3. Differential Scanning Calorimetry (DSC)

Differential scanning calorimetry was performed in order to study the thermal transitions of the composites. Samples of 5 ± 0.1 mg sealed in aluminum pans were used in a PerkinElmer Pyris Diamond DSC differential scanning calorimeter (PerkinElmer, Solingen, Germany) calibrated with pure indium and zinc standards. The system included a PerkinElmer Intracooler 2 (PerkinElmer, Solingen, Germany) cooling accessory. Each sample was heated to 280 °C to erase previous thermal history and then cooled to room temperature at a rate of 20 °C/min under a nitrogen atmosphere. Then, a second heating cycle to 280 °C at the same scanning rate was performed to obtain the melting temperature (T_m_). The degree of crystallinity (X_c_) was calculated using Equation (2) [41,42]:

(2) $$X_{c}\;(\%)=\frac{{\Delta H}_{m}}{\left(\frac{100-wt}{100}\right){\Delta H}_{m}^{0}}\;\times \;100\%,$$

where ${\Delta H}_{m}^{0}$ = 140 J/g, the heat of fusion of 100% crystalline PET [43,44], and ΔH_m_ is the experimental heat of fusion of each sample.

#### 2.2.4. Polarized Light Microscopy (PLM)

The crystallization behavior of the samples was investigated by polarized light microscopy (PLM). After melting, the samples were cooled at a rate of 10 °C/min, and optical micrographs were recorded using a polarizing light microscope (Nikon, Optiphot-2, Tokyo, Japan) equipped with a Linkam THMS 600 heating stage (Linkam Scientific Instruments, Tadworth, UK), a Linkam TP 91 control unit (Linkam Scientific Instruments, Tadworth, UK), and a Jenoptic ProgRes C10Plus camera (Jena, Germany) with the Jenoptik ProgRes CapturePro software.

#### 2.2.5. Thermogravimetric Analysis (TGA)

Thermogravimetric analysis of the rPET nanocomposites was carried out using a SETARAM SETSYS TG-DTA 16/18 instrument (SETARAM, Caluire, France). Samples (4.5 ± 0.5 mg) were placed in appropriate alumina crucibles, while an empty alumina crucible was used as a reference. All samples were heated from ambient temperature to 600 °C in a 50 mL/min flow of N_2_, with a heating rate of 10 °C/min, while continuous recordings of sample temperature, sample mass, and its first derivative were taken. T_0.5%_ (the temperature at which 0.5% weight loss was achieved) and T_max_ values (the temperature corresponding to the maximum degradation rate) were calculated.

#### 2.2.6. Pyrolysis–Gas Chromatography/Mass Spectrometry (Py–GC/MS)

For Py–GC/MS analysis of the neat rPET and rPET/BC5 nanocomposites, a very small amount of each material (≈1 mg) was “dropped” initially into the “Double-Shot” EGA/PY 3030D Pyrolyzer (Frontier Laboratories Ltd., Fukushima, Japan) using a CGS-1050Ex carrier gas selector. The sample was subjected to flash pyrolysis at 500 °C, selected according to the TGA results and the maximum degradation temperature. The GC oven temperature was programmed from 50 °C (2 min) to 200 °C at 5 °C min^−1^ (8 min hold), followed by heating to 300 °C at 20 °C min^−1^ (5 min hold). The pyrolysis products were separated using an Ultra ALLOY^®^ capillary column (30 m × 0.25 mm i.d., 5% diphenyl/95% dimethylpolysiloxane stationary phase). Helium was used as the carrier gas at a flow rate of 1 mL min^−1^ with a split ratio of 1:50. Mass spectra were acquired in the *m*/*z* range of 40–500 amu with an interface temperature of 300 °C and an ion source temperature of 200 °C. Compound identification was performed using NIST11 and Frontier F-Search 4.3 libraries, considering matches with a similarity index higher than 80%.

#### 2.2.7. Scanning Electron Microscopy (SEM)

The cryo-fractured surfaces of the rPET/BC nanocomposites were examined using a JEOL JMS 7610F (Freising, Germany) scanning electron microscope equipped with an Oxford AZTEC ENERGY ADVANCED X-act EDS detector (Freising, Germany). Imaging was performed at an accelerating voltage of 5 kV. Prior to placement in the vacuum chamber, the specimens were sputter-coated with carbon to ensure adequate electrical conductivity.

#### 2.2.8. Broadband Dielectric Spectroscopy (BDS)

The dielectric permittivity and molecular dynamics of the polymers were assessed by BDS [45] under a nitrogen atmosphere and in the temperature range between −140 and 140 °C, employing a Novocontrol BDS setup (Novocontrol GmbH, Montabaur, Germany). The samples were in the form of cylindrical disks, with a thickness of 1–2 mm and a diameter of 20 mm, and were measured as received, i.e., without any special thermal treatment. To evaluate the dielectric response, the disks were placed between finely polished disk-like electrodes made of brass, forming a sandwich-like capacitor. The complex dielectric permittivity, ε* = ε′ − i·ε′′, was recorded as a function of frequency in the range of 10^−1^ to 10^6^ Hz, isothermally at the various temperatures, with increasing steps of 5 or 10 K.

#### 2.2.9. Intrinsic Viscosity (IV) Measurements

The intrinsic viscosity of the prepared materials was measured with a Ubbelohde viscometer (Schott Gerate GMBH, Hofheim, Germany) at 25 °C. The samples were dissolved in a 60/40 *w*/*w* solution of phenol and tetrachloroethane, selected to effectively dissolve PET, by heating at 80 °C for 15 min. Solutions were prepared at a concentration of 1 mol/L. Once cooled, each sample was passed through a disposable 0.2 μm Teflon membrane filter to remove any potential solid residues. Nevertheless, complete elimination of nanoparticles could not be fully ensured; therefore, the reported values for the nanocomposite samples should be considered approximate. These values were calculated according to the Solomon–Ciuta Equation (3) of a single-point measurement:

(3) $$\left[\eta \right]\;=\;\frac{{\left[2\left\{\frac{t}{t_{0}}-\;ln\left(\frac{t}{t_{0}}\right)-1\right\}\right]}^{1/2}}{c},$$

where c is the solution concentration, t is the flow time of the solution, and t_0_ is the flow time of the solvent. The discussed values are the average of three measurements.

The intrinsic viscosity [η] measurements were subsequently employed to determine the viscosity-average molecular weight (Mv) of the rPET materials, using the Berkowitz Equation (4) [46], as adapted in earlier studies [47]:

(4) $$Mv\;=\;3.29\times 10^{4}{\left[\eta \right]}^{1.54}.$$

## 3. Characterization and Discussion

### 3.1. Structural Characterization

**Figure 1:**
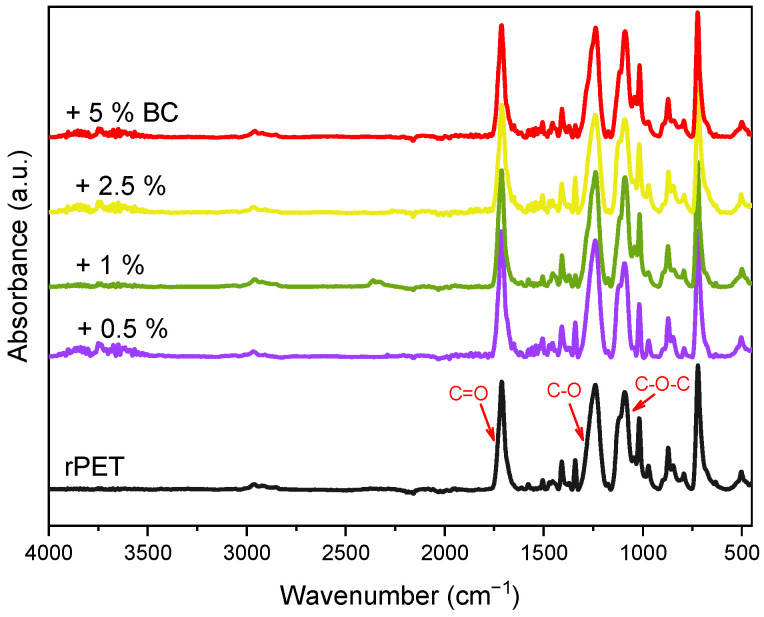
IR spectra of neat rPET and its biochar-filled nanocomposites at different filler loadings.

The ATR spectrum of neat rPET (Figure 1) presents a well-preserved chemical structure, with strong absorption peaks at 1713 cm^−1^ (C=O stretching of ester groups), 1238, and 1090 cm^−1^ (C–O stretching), confirming the integrity of the polyester backbone. Aromatic C=C stretching is detected at 1577 and 1504 cm^−1^, while a weak O–H stretch at around 3300 cm^−1^ suggests minor hydrolysis or surface oxidation, typical in post-consumer PET [48,49].

As for the nanocomposite materials, spectra remain basically identical to that of neat rPET, with no new absorption peaks nor detectable shifts, confirming that the incorporation of BC does not alter the chemical structure of the rPET (Figure S1). This note is in line with previous studies on polymer-based composites, where the absence of new bands is considered as proof of no specific chemical bonding and the dominance of physical interactions between the additive and the polymer matrix [50,51].

**Figure 2:**
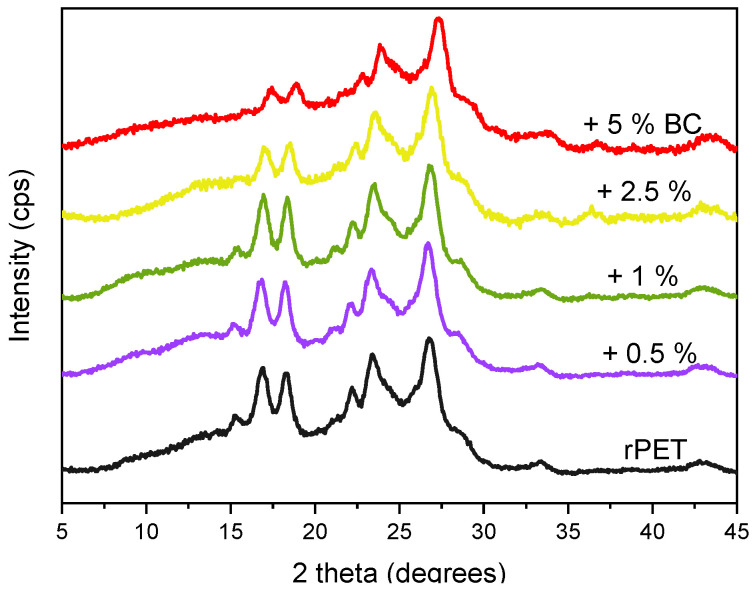
XRD spectra of neat rPET and its biochar-filled nanocomposites at different filler loadings.

The crystalline structure of the nanocomposite materials was studied using XRD patterns. In Figure 2, the neat rPET presents characteristic diffraction peaks at approximately 2θ = 16.8, 18.2, 22.2, 23.4, and 26.5° that are attributed to the crystalline planes of semicrystalline PET [52,53,54]. No new diffraction peaks were observed upon the incorporation of BC, indicating that no additional crystalline phases were formed. At higher BC loadings (2.5 and 5% wt.), a slight decrease in peak intensity and minor peak broadening were observed, suggesting a structural disorder of the crystalline phase.

Consequently, XRD analysis indicates that BC in higher ratios interferes with the development of well-organized crystalline domains, leading to smaller and less perfect crystallites and, thus, an increased amorphous halo. Similar trends have been reported in polymer composites with carbonaceous additives, where increasing the nucleating agent is accompanied by broader diffraction peaks [55,56]. The preservation of the characteristic PET crystalline structure, together with the absence of additional crystalline phases, indicates that biochar acts primarily as a physical modifier of the rPET matrix. This observation is consistent with the FTIR findings and provides a structural basis for interpreting the thermal degradation and UV-aging behavior discussed in the following sections. The calculated degree of crystallinity is presented in Table 2 and is discussed below.

### 3.2. Thermal Characterization

**Figure 3:**
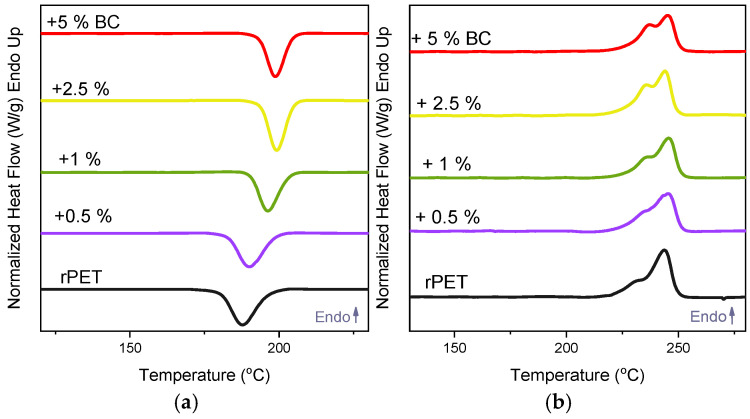
DSC thermograms of neat rPET and its biochar-filled nanocomposites during (**a**) cooling and (**b**) heating scans.

The DSC cooling and heating thermograms of neat rPET and its nanocomposites with BC are shown in Figure 3. During cooling (Figure 3a), neat rPET exhibits a crystallization peak at around 187 °C, which is typical of the polymer’s ability to recrystallize. Incorporation of BC shifts the crystallization peak to slightly higher temperatures and sharpens the transition, indicating that biochar particles act as effective heterogeneous nucleating agents, facilitating faster crystal formation during cooling [27]. Similar nucleation-promoting effects of biochar have been reported in other polymer/biochar systems, where the filler provides additional heterogeneous crystallization sites and modifies crystallization kinetics [57].

During the subsequent heating scans (Figure 3b), neat rPET displays a melting endotherm with a noticeable shoulder, indicative of imperfect or multiple crystal populations—a common feature in recycled polymers due to chain heterogeneity. This double melting phenomenon becomes more pronounced in the nanocomposites, suggesting that BC influences lamellar reorganization: the less stable crystals formed during cooling recrystallize into more perfect structures upon heating, producing distinct melting endotherms [58]. These results are in total agreement with the XRD analysis, where peak broadening suggested reduced crystal perfection in the presence of BC [59]. It is also important to note that no cold crystallization (T_cc_) was observed, since the samples had already crystallized during the preceding cooling step.

Regarding crystallinity, the calculated X_c_ (DSC) values (~25–28%) remained essentially unchanged across all samples (Table 2), suggesting that BC predominantly affects crystallization kinetics and melting behavior rather than the final crystalline fraction. In agreement with the DSC results, the XRD-derived crystallinity values X_c_ (XRD) exhibit only a slight decrease, from 38% for neat rPET to 34% for the highest BC loading, confirming that the incorporation of BC does not significantly alter the overall crystalline structure of the polymer. This minor reduction can be attributed to a partial restriction of chain mobility and crystal growth at higher filler contents [60].

**Figure 4:**
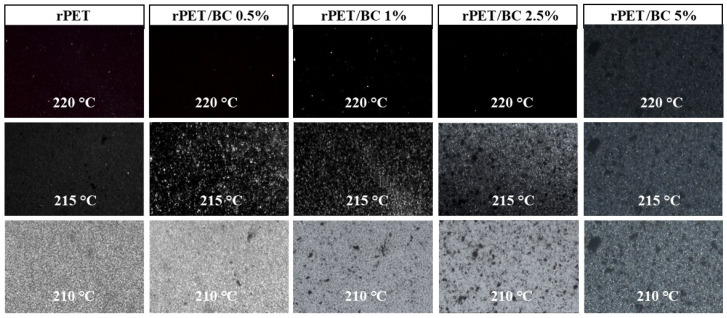
PLM images of neat rPET and its biochar-filled nanocomposites at different biochar loadings, recorded at 220, 215, and 210 °C during cooling from the melt phase.

The polarized light microscopy (PLM) images (Figure 4) further support the nucleating effect of BC on the crystallization of rPET and complement the conclusions suggested by the DSC results. At 220 °C, neat rPET remains basically in the melt state with no visible spherulites formed, while the nanocomposite containing 5% wt. BC already shows the onset of crystal formation, confirming that BC promotes earlier crystallization upon cooling. As the BC ratio rises, the density of spherulites noticeably increases and their size remains comparably small, indicating a high nucleation density and limited spherulitic growth, in line with the sharper and slightly upshifted crystallization peak noticed in DSC.

At 215 °C, neat rPET starts to form a small number of spherulites, while all composites reveal an advanced crystalline morphology, with several spherulites distributed within the polymer matrix. Finally, at 210 °C, all samples have almost completed crystallization, presenting a dense network of small spherulites. These findings clearly demonstrate that sewage sludge-derived BC accelerates the crystallization process, without changing the final degree of crystallinity X_c_ values (~21–23%) calculated by DSC [61].

In addition, the PLM images confirm a uniform dispersion of BC throughout the matrix, since no large agglomerates interrupt the spherulitic texture. This note is in agreement with the SEM micrographs, discussed in the following section, where BC particles seem to be efficiently dispersed.

**Figure 5:**
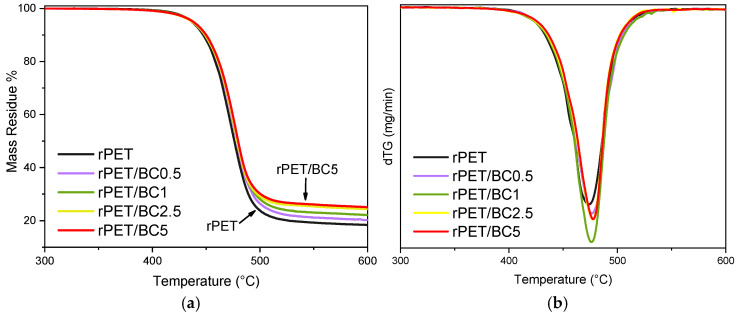
TGA thermograms of neat rPET and its biochar-filled nanocomposites: (**a**) mass loss and (**b**) dTG.

The thermal degradation behavior of rPET nanocomposites was evaluated by TGA at a heating rate of 10 °C/min under a nitrogen atmosphere. The mass loss graph and the derivative thermogravimetry (dTG) curve are shown in Figure 5.

The mass loss curve (Figure 5a) presents stability up to 386 °C, with the main degradation occurring in a single step in the range of 386–540 °C, leaving a residual mass of 18.4%. These values are consistent with the typical thermal degradation behavior of rPET [62]. The incorporation of BC results in a slight reduction in the onset temperature to around 386 °C (Table 2). This earlier initiation of degradation could be linked to oxygen-containing groups or inorganic ash that are present on the BC surface and can promote the initial chain scission reactions of rPET. At the same time, the main degradation step is slightly shifted toward higher temperatures, indicating that the inclusion of BC can enhance the thermal stability of the matrix. The dTG curves (Figure 5b) further support this observation, as the maximum degradation rate temperatures are marginally shifted toward higher values compared with neat rPET. This observation suggests that the BC particles hinder the progression of degradation, likely through a barrier effect that restricts heat transfer within the polymer matrix [63]. In addition, the residual mass progressively increases from 18.4 to 25% for the neat rPET and rPET/BC5, respectively, due to the thermally stable carbonaceous nature of biochar, resulting in enhanced char residue formation [64]. The increased char residue, together with the slight shift in T_max_ toward higher temperatures, suggests that BC contributes to the stabilization of rPET during thermal degradation [65].

**Figure 6:**
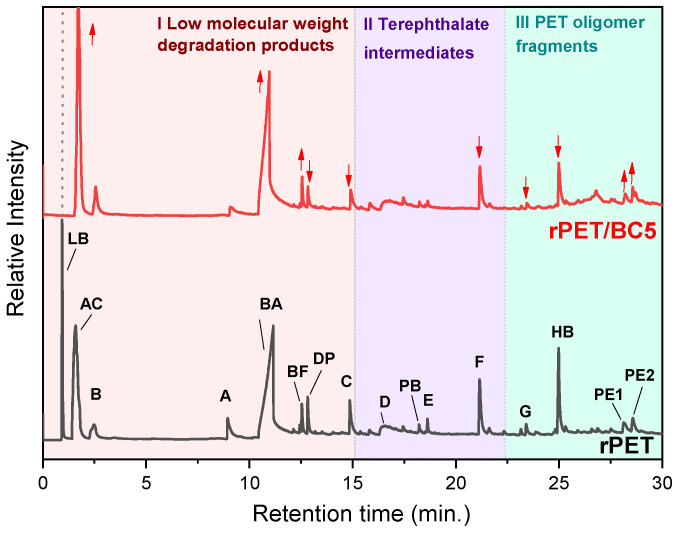
Total ion chromatograms (TICs) of neat rPET and rPET/BC5, with the annotated compounds. The red arrows indicate pyrolysis products whose relative peak intensities increase or decrease in the rPET/BC5 nanocomposite compared with neat rPET.

With the aim of gaining further insight into the thermal degradation mechanism of the prepared materials, Py–GC/MS analysis was performed on neat rPET and rPET/BC5. The total ion chromatograms (TICs) and the corresponding assignments of the major degradation products are presented in Figure 6, whereas the assigned compounds as determined from their MS spectra, together with their relative abundances (a.u.), are summarized in Table 3. The chromatographic profiles of both neat rPET and rPET/BC5 are dominated by characteristic PET degradation products, including benzoic acid (peak BA, Rt = 11.06 min), vinyl benzoate (peak A, Rt = 8.96 min), divinyl terephthalate (peak C, Rt = 14.86 min)), 4-(vinyloxycarbonyl)-benzoic acid (peak D, Rt = 16.49 min), ethan-1,2-diyl dibenzoate (peak E, Rt = 18.64 min), and higher-molecular-weight terephthalate oligomer fragments (peaks F–PE_2_, Rt = 21.17–28.6 min). The above mentioned products originate mainly from ester bond cleavage, decarboxylation, and transesterification reactions taking place during the thermal decomposition of PET, facilitated by pyrolysis conditions. Acetaldehyde (peak AC, Rt = 1.59 min) and benzoic acid were identified as the most abundant degradation products, in agreement with previous reports on PET pyrolysis [32,51].

For discussion purposes, the identified products were classified into three groups: (i) low-molecular-weight degradation products, (ii) terephthalate-derived intermediates, and (iii) higher-molecular-weight PET oligomer fragments (Figure 6). The overall similarity of the chromatographic profiles confirms that the incorporation of BC does not introduce new degradation pathways or alter the fundamental decomposition chemistry of PET. This observation is consistent with the FTIR results, where no new absorption bands were detected, indicating the absence of significant chemical interactions between the polymer matrix and the filler. Nevertheless, notable differences in the relative abundance of individual degradation products were observed. In particular, a notable difference between the chromatographic profiles of neat rPET and rPET/BC5 is the absence of the CO_2_ and vinyl benzoate peaks in the BC-containing nanocomposite. CO_2_ is typically produced through decarboxylation reactions occurring during PET thermal degradation, while vinyl benzoate is considered a characteristic low-molecular-weight product generated by ester bond cleavage and chain scission processes. The absence of these compounds in the rPET/BC5 chromatogram may imply that the incorporation of BC suppresses specific degradation pathways, leading to the formation of volatile decomposition products.

This interpretation is further supported by the distribution of the detected pyrolysis products. Specifically, the ratio between low-molecular-weight degradation products (Rt < 15 min) and higher-molecular-weight PET oligomer fragments (Rt > 15 min) decreased from 1.03 for neat rPET to 0.83 for rPET/BC5, corresponding to approximately a 19% reduction. Such behavior may indicate that BC suppresses secondary fragmentation reactions and promotes the retention of larger terephthalate-containing species during thermal decomposition. These findings are in excellent agreement with the TGA results presented previously, where the nanocomposites exhibited slightly higher T_max_ values together with increased residual char formation. The higher char yield observed by TGA, combined with the lower abundance of volatile degradation products detected by Py–GC/MS, suggests that the carbonaceous biochar particles act as a physical barrier and adsorption medium, restricting the evolution of degradation volatiles and slowing the progression of thermal decomposition [66].

### 3.3. Scanning Electron Microscopy (SEM)

**Figure 7:**
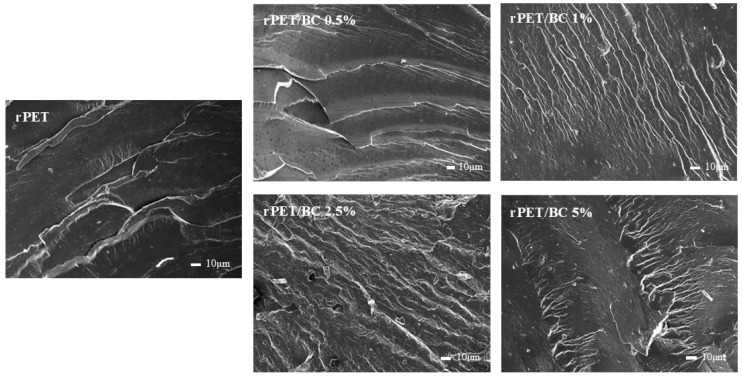
SEM micrographs of neat rPET and its biochar-filled nanocomposites at different filler loadings.

The micromorphology of the nitrogen cryo-fractured cross-section of nanocomposites (Figure 7) further supports the good interfacial compatibility between the filler (Figure S2) and the polymer. All the materials show a continuous surface with no visible gaps, voids, or pull-out phenomena. These observations are typically linked to strong interfacial bonding and coherent stress transfer from the rPET matrix to the BC, as reported for other biochar-filled polymer systems [50,67].

Neat rPET and low-ratio nanocomposites (0.5 and 1% wt.) present a comparatively smoother surface with multiple crack planes, indicative of a brittle yet homogeneous morphology, which is consistent with finely dispersed fillers that do not introduce large stress concentrations. On the other hand, higher ratios (2.5 and 5% wt.) introduce a rougher surface, reflecting the increased population of rigid particles which locally disturb crack propagation. Still, the absence of interfacial voids suggests that the mechanical properties might not be significantly downgraded.

Concerning the filler dispersion, the rPET/BC0.5 exhibits well-distributed BC particles within the matrix, indicating effective dispersibility during mixing. For higher BC ratios, the dark color of the filler dominates, making individual particles unobservable. However, the absence of large aggregates or phase separations points to a macroscopically homogeneous dispersion [23]. These SEM findings are in line with the PLM results, as depicted in Figure 4, highlighting that good dispersion and interfacial adhesion are key factors for functional enhancement of the nanocomposites.

### 3.4. Dielectric Permittivity and Molecular Mobility (BDS)

**Figure 8:**
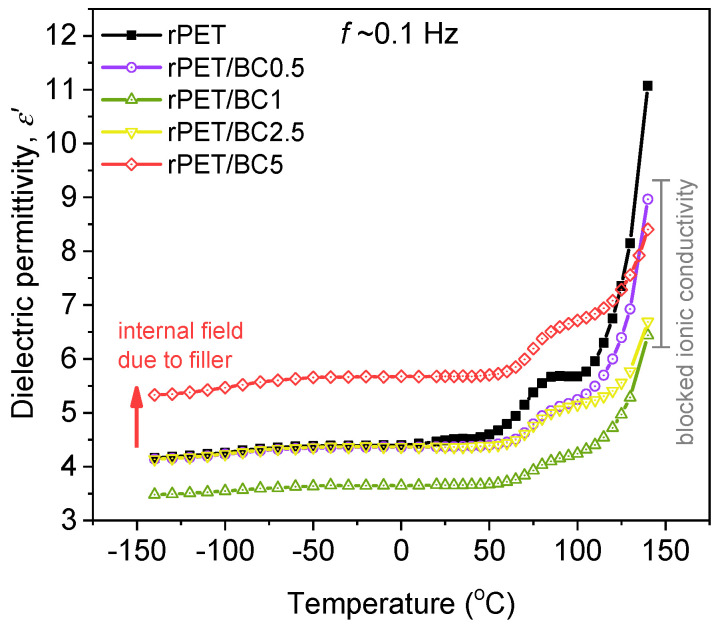
The temperature dependence of the real part of the dielectric permittivity, ε′, at the selected low frequency of 0.1 Hz, for all samples.

In Figure 8, the real part of dielectric permittivity, ε′ (traditionally called the ‘dielectric constant’), against temperature is presented in the form of isochronal plots. ε′ exhibits an expected increasing trend with temperature. At lower temperatures, below T_g_, wherein only dipolar contributions contribute to the signal, ε′ varies between 3.5 and ~7, as expected for insulating polymers [45]. At the lowest temperatures, for 0–2.5% wt. BC, ε′ exhibits low values (up to ~4), whereas for the highest BC loading of 5% wt., the signal exhibits general elevated values (~5.5). This possibly suggests the formation of a stronger internal field due to the existence of BC particles, which are characterized by increased conductivity (carbon-based fillers) compared to the polymer [68,69]. The effect could be indicative of the sufficiently good dispersion of the BC particles, facilitated also by the increasing number of particles and the simultaneous decrease in the distance between them.

On the other hand, at the highest temperature side in Figure 8, i.e., well above *T*_g_, *ε*′ increases sharpy due to the involvement of ionic conductivity arising from free charge/ion transport throughout the polymer matrix. Interestingly, the ionic conductivity is high in neat rPET and decreases significantly in the nanocomposites. Since the polymer was found to be amorphous (no crystal existence), the suppressed conductivity in the composites suggests the severe blocking of the ions at the BC surfaces [45]. This is an additional, although indirect, proof of good BC dispersion, that is in agreement with the SEM micrographs (Figure 7).

**Figure 9:**
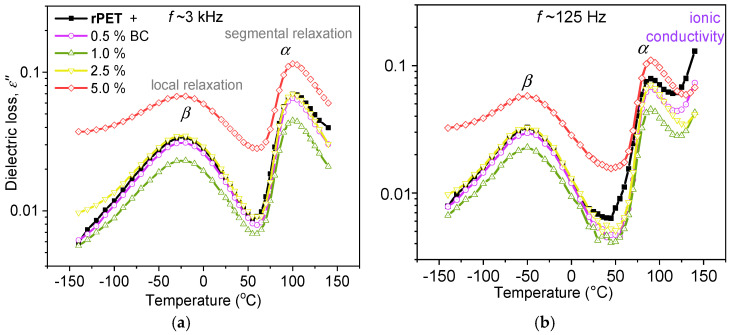
Comparative isochronal plots of ε″ for all samples, being shown at two selected frequencies, (**a**) 3 kHz and (**b**) 125 Hz. Indicated are the recorded local and segmental relaxations.

Coming to molecular dynamics, attention is turned to the imaginary part of the dielectric permittivity, *ε*″, that is considered to express the dielectric loss. The temperature dependence of *ε*″ is presented in Figure 9, at the two selected frequencies of 3 kHz (Figure 9a) and 125 Hz (Figure 9b). Therein, and at temperatures below and closely above *T*_g_, *ε*″ is dominated by the relaxation of dipole moments associated with local and segmental motions of rPET. The latter are recorded as *β* and *α* relaxations, respectively.

The local *β* relaxation is known in PET. Previous measurements of conventional PET of various crystallinity degrees [70,71,72] have shown that *β* relaxation is activated solely within the amorphous phase [70]. *β* relaxation has been attributed to torsional vibrations of the main chain correlated over a length corresponding to one monomer [73,74]. Moreover, it has been proposed that *β* originates from carbonyl-driven localized motions of carbonyl and phenyl groups [71].

At higher temperatures, along with the general increase in the signal, the so-called *α* relaxation is recorded. It is widely accepted that *α* relaxation is associated with the glass–rubber transition; in other words, it is the dielectric analog of the glass transition [45]. The *α* process is believed to arise from the relaxation of dipole moments, being perpendicular on the polymer backbone/axis [45,75].

At first glance, in Figure 9, it seems that there are no significant variations in the *β* and *α* relaxation in the rPET/BC nanocomposites. To extract more in-depth information, analysis of the complex BDS spectra was performed [76,77,78]. In particular, the following mathematical equations were employed. The Havriliak–Negami (HN) function [79] (Equation (5)) was employed to fit the asymmetric peaks—in our case, the amorphous *α* relaxation.

(5) $$\varepsilon *\left(f\right)=\frac{\Delta \varepsilon }{{\left[1+{\left(\frac{if}{f_{0}}\right)}^{a_{HN}}\right]}^{\beta_{HΝ}}}+\varepsilon_{\infty },$$

where *f*_0_ is a characteristic frequency related to the frequency of maximum dielectric loss, *ε_∞_* is a value of the real part of the dielectric permittivity (*ε*′) at *f* >> *f*_0_, *α*_HN_ is a shape parameter that denotes the width of the relaxation time range, and *β*_HN_ is another shape parameter that evaluates the symmetry of the ε″ (*f*) peak. Δ*ε* is the dielectric strength (~magnitude of the peaks) or the corresponding contribution of the dipoles to the dielectric permittivity. The same model can be fitted for symmetric peaks, fixing the asymmetricity parameter *β*_HN_ as 1 and, thus, becomes the so-called Cole–Cole equation [45]. To simulate the conductivity-related contributions at the higher frequency side of the measurement window, the additional linear term ‘$-i\cdot \sigma_{0}/{\left(2\pi f\right)}^{Z}\varepsilon_{0}$’ was employed in Equation (5) [45].

**Figure 10:**
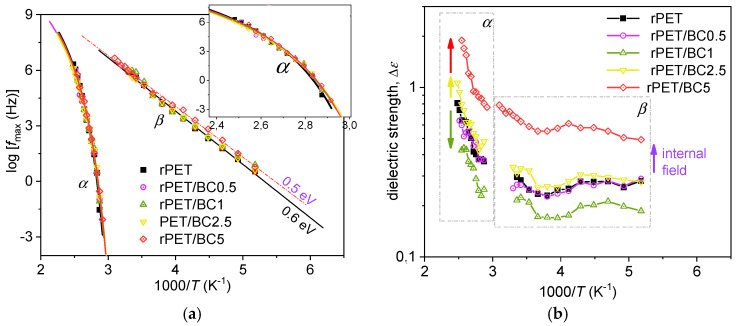
(**a**) The Arrhenius plots for the local β and segmental α relaxation. The straight and curved lines connecting the experimental points are fittings of the Arrhenius and the Vogel–Tammann–Fulcher–Hesse equations, respectively. (**b**) The reciprocal temperature dependence of the dielectric strength for both relaxations.

From the results of the analysis, the Arrhenius and the Δ*ε* plots were constructed. The plots are presented in Figure 10; more specifically, in terms of time scale in Figure 10a, and the reciprocal temperature dependence of Δ*ε* in Figure 10b.

Upon the construction of the Arrhenius plots, the Arrhenius (straight line) and the Vogel–Fulcher–Tammann–Hesse (VFTH, curved lines) [72,80] equations, i.e., Equations (6) and (7), were fitted to the local *β* and segmental *α* relaxations, respectively. In Equation (6), *f*_0,Arrh_ is a pre-exponential factor and *E*_act_ is the activation energy. In Equation (7), *f*_0,VFTH_ is a frequency constant, *T*_0_ is the Vogel temperature at which *f*_0,VFTH_ → 0, and *D* is the so-called fragility strength parameter [80].

(6) $$f\left(T\right)=f_{0,Arrh\;}\cdot e^{-\frac{E_{act}}{kT}}.$$

(7) $$f\left(T\right)=f_{0,VFTH\;}\cdot e^{-\frac{{DT}_{0}}{T-T_{0}}}.$$

In Figure 10a, the secondary *β* relaxation (*α*_HN_~0.3–0.4, *β*_HN_ = 1 in Equation (5)) exhibits the expected linear trend for local dynamics, obeying the Arrhenius equation. The *E*_act_ for almost all systems equals 0.6 eV. The results are consistent with previous findings on PET [71]. In the case of rPET/BC5, *β* exhibits a slight acceleration and a mild drop in the *E*_act_ (= 0.5 eV). The Δ*ε*(*Τ*) trends for *β* increase in Figure 10b, as expected for local relaxations [45].

In the case of *α* relaxation, the process was fitted with HN terms of *α*_HN_~0.3 and *β*_HN_ = 1, in general, for all samples. This suggests a symmetric relaxation process with wide relaxation time range, regarding segmental dynamics. It should be recalled that in the case of amorphous unconstrained mobility, the respective *α* relaxation is usually expected to be asymmetric and relatively narrow [45], whereas the Δ*ε*(*Τ*) is expected to decrease. Despite the amorphous character of rPET here, there are opposite results, e.g., the increasing Δ*ε*(*Τ*) in Figure 10b. The time scale of *α* relaxation is as expected, namely a curved trend in Figure 10a (and the inset). This is characteristic of cooperative dynamics being mathematically expressed via the VFTH equation (Equation (7), curved lines).

Thus, from the fittings of VFTH to the experimental points of *α* and by extrapolating the lines to the equivalent frequency of DSC (i.e., *f*_eq,DSC_~10^−2.8^ Hz related to the corresponding *τ*_rel,DSC_~100 s), the so-called dielectric glass transition temperature, *T*_g,diel_, was evaluated. Regarding the cooperative relaxations, the fragility index, *m*_α_, was evaluated as well. Practically, this was done by fixing *f*_0,VFTH_ to the phonon frequency, *f*_phonon_~10^13^ Hz, and then by estimating *D* and calculating *m*_α_ via Equation (8) [81].

(8) $$m_{a}=16+590/D.$$

**Figure 11:**
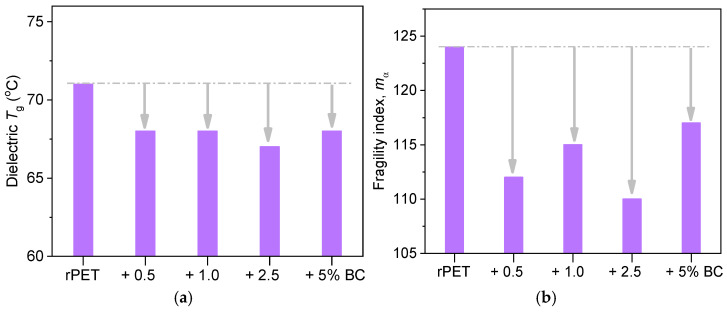
Nanocomposite composition effects on the (**a**) dielectric T_g_ and (**b**) fragility index of α relaxation, m_α_.

The *T*_g,diel_ is shown in Figure 11a to decrease from 71 °C (neat rPET) to 67–68 °C with the addition of the BC particles. The effect suggests a mild acceleration of segmental mobility, or easier chain diffusion in the composites. In Figure 11b, the fragility index *m*_α_ values are presented. *m*_α_ equals 124 for rPET and, interestingly, drops to 110–117 in the rPET/BC nanocomposites. The lowest values are recorded for 0.5–2.5% BC loadings. The drop in *m*_α_ has been correlated with a decrease in the chain–chain cooperativity level; moreover, it may indicate an increase in the cooperativity length, *ξ* [82]. It is expected that the glass transition of the polymer takes place in bulk-like regions, namely, not close to the BC particles. To rationalize together the mild *T*_g,diel_ decrease and the moderate drop in *m*_α_, the following scenario can be speculated, as employed for polymer nanocomposites in the past [83,84]. Within the nanocomposites, rPET chains should be condensed around the BC entities and, thus, the polymer density of bulky rPET (away from the BC) must be lower. This is compatible with the increased free volume away from the particles, which is expected to lead to less constrained and less cooperative chain mobility. Obviously, these are strong statements and require further experimental support.

### 3.5. Resistance to UV-Induced Degradation

The effect of sewage-derived BC on the resistance of nanocomposites to UV-induced degradation was evaluated through accelerated weathering tests. UV exposure is known to promote the photo-oxidation of polyesters, leading to chain scission and a consequent drop in molecular weight. In recycled PET systems, photo-oxidative degradation is particularly important because molecular weight loss is directly associated with deterioration of mechanical performance, reduced service lifetime, and increased susceptibility to embrittlement and fragmentation during long-term environmental exposure. Therefore, intrinsic viscosity (IV) measurements were performed before and after 90 days of UV aging in order to assess the stabilizing effect of the additive. The IV values [η] of neat rPET and its nanocomposites, along with the corresponding molecular weights (Mv), are presented in Table 4.

Before UV aging, all nanocomposites demonstrate comparable values. The neat rPET shows an IV value of 0.49 dL/g, corresponding to an Mv of approximately 10.9 kg/mol. Upon incorporation of low BC loadings, a slight increase is observed, reaching 0.51 and 0.55 dL/g for the nanocomposites containing 0.5 and 1% wt. BC, respectively. This increase can be attributed to the branching reactions that may occur between anhydride groups of PMDA and functional groups on the BC surface. However, at higher BC loadings, a decrease in IV is observed, reaching the values of neat rPET. This trend suggests that BC at these ratios could possibly hinder branching reactions or even promote competing degradation mechanisms [85].

**Figure 12:**
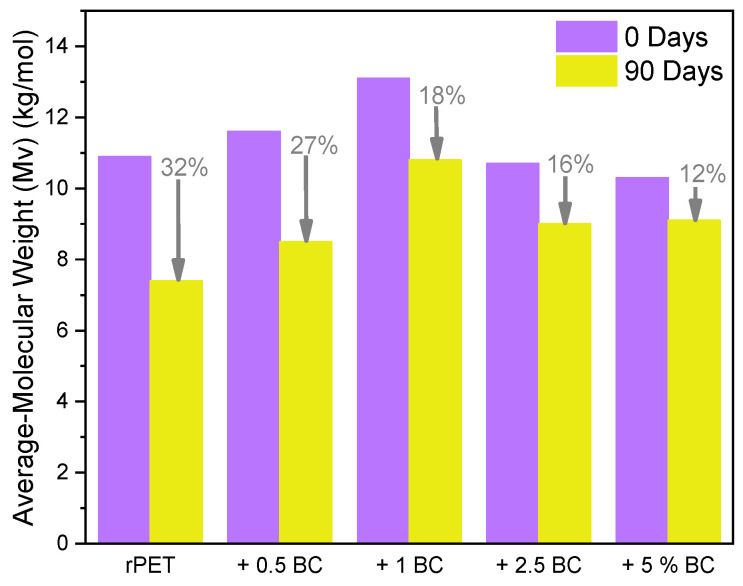
Percentage reduction in average molecular weight (Mv) of rPET and its biochar-filled nanocomposites after 90 days of UV irradiation.

Upon 90 days of UV irradiation, all nanocomposites displayed a reduction in IV and Mv values, indicating that photooxidation occurred, leading to chain scission reactions, as expected (Figure 12). The rPET sample showed the highest decrease in Mv (32%), while the reduction gradually decreased with increasing BC loading, reaching 12% for rPET/BC5. The progressive improvement in Mv retention demonstrates that BC protects the polyester matrix against UV-induced degradation. The improved molecular weight retention indicates that BC effectively limits photo-induced chain scission reactions, thereby preserving the integrity of the polyester backbone during prolonged UV exposure. This effect can probably be attributed to the carbonaceous structure that is capable of absorbing part of the UV irradiation, hindering the formation of radicals and reducing chain scission reactions [15,28,29].

**Figure 13:**
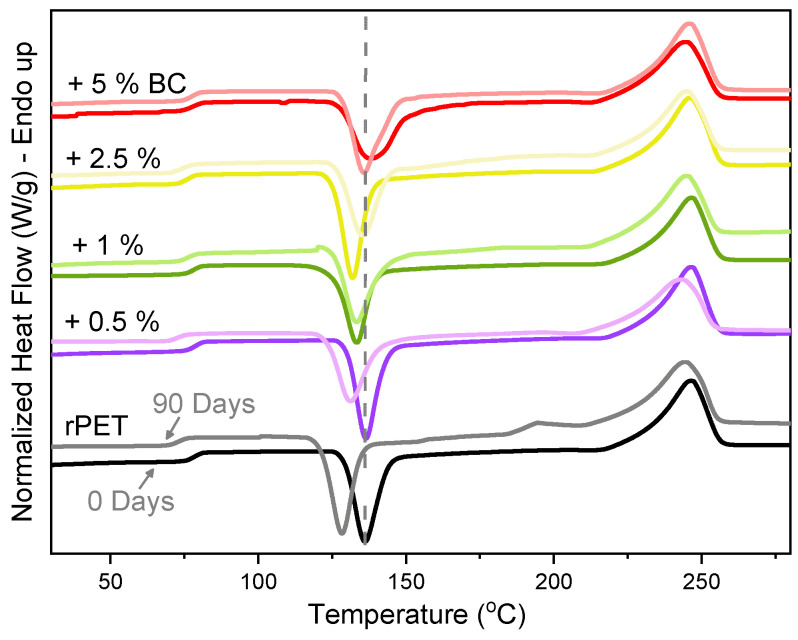
DSC thermograms of quenched rPET and its biochar-filled nanocomposites before and after 90 days of UV aging.

To further explore the effect of UV irradiation on the thermal properties of nanocomposites, DSC measurements were performed before and after 90 days of UV exposure. In order to evaluate the amorphous phase, without differences in thermal history and crystallinity, the samples were melted above the T_m_ and rapidly cooled in liquid nitrogen. The thermograms recorded during the subsequent heating scan are presented in Figure 13. Overall, only minor changes were observed in thermal transition after the irradiation.

Upon UV irradiation, neat rPET exhibits a decrease in both T_g_ and T_cc_ temperatures, from 78 to 73 °C and from 136 to 128 °C, respectively (Table 4). This observation suggests increased chain mobility, which can be linked to lower molecular weight due to photo-oxidation. Additionally, the aged rPET sample shows a secondary melting endotherm at 194 °C, probably indicating the formation of less perfect crystals during degradation. On the other hand, the decrease in these thermal events is less pronounced in nanocomposites, suggesting that BC limits the chain scission reactions caused by UV aging. This observation is consistent with IV measurements, which demonstrate a significantly shorter Mv range of loss for the BC-containing samples compared to neat rPET. Therefore, DSC results additionally support the beneficial role of BC as a UV stabilizer as it is capable of absorbing part of UV irradiation and preserving the structural integrity of the polymer matrix [28]. When considered together with the TGA and Py–GC/MS results, the UV-aging experiments suggest that sewage sludge-derived biochar improves the resistance of rPET against both thermal and photo-oxidative degradation. The observed reduction in molecular weight loss, combined with the moderation of thermal degradation pathways, highlights the potential of biochar as a multifunctional stabilizing additive capable of extending the service lifetime of recycled PET materials.

## 4. Enviromental and Societal Impact

The development of rPET/BC nanocomposites represents a systemic transformation from a linear “take–make–dispose” model toward a circular economy. By repurposing municipal sewage sludge to extend the life-cycle of post-consumer plastic, this research addresses two critical waste management streams and provides a tangible pathway for a “double-circular” economy.

### 4.1. Environmental Implications

From an environmental perspective, the use of rPET is a critical alternative to virgin polymer production, which is inherently energy demanding. Producing 1 kg of plastic material requires between 62 and 108 MJ of energy and accounts for more than 4% of global oil and gas consumption [86]. However, the use of plastic insulation materials can save more than 140 times the energy needed for their production throughout their service life [87].

Since 1950, approximately 8300 million tons of plastic have been made, resulting in 6300 million tons of plastic waste being thrown into landfill or dispersed into the environment [86]. Currently, about 415 million tons of plastic are produced annually worldwide. Between 1950 and 2015, 79% of plastic waste was reported as poorly managed, leading to a cumulative 5 billion tons of plastic waste currently residing in landfills or the natural environment [86].

Regarding the sewage sludge biochar used in this study, the transformation of municipal sewage sludge into biochar offers a strategic solution to the global increase in wastewater residues, which currently exceed 13 million tons annually in Europe alone [88]. Sewage sludge management remains a significant environmental challenge due to its high volume and the accumulation of contaminants such as heavy metals, pathogens, pharmaceuticals, and microplastics. Inadequate treatment or disposal can lead to soil and water contamination, human health risks, and secondary pollution pathways. Developing safe and effective treatment and valorization strategies is therefore critical to reduce environmental threats, ensure regulatory compliance, and transform sewage sludge from a waste liability into a resource within circular economy frameworks [89,90]. Pyrolysis represents a promising and safe pathway for transforming sewage sludge into biochar suitable for secondary-life applications. This thermochemical process stabilizes organic matter, reduces pathogen content, and immobilizes hazardous contaminants, thereby minimizing environmental risks. As a result, sewage sludge biochar can be safely valorized in advanced material and circular economy applications [91].

### 4.2. Societal Impact

From a societal perspective, this research supports the transition toward a circular economy by providing municipalities with value-added applications for sludge, potentially offsetting management costs and promoting local industrial symbiosis. Linking wastewater treatment facilities, recycling industries, and advanced manufacturing sectors creates new value chains that can stimulate innovation and support green jobs [92,93].

Moreover, as these materials (PET and biochar) are compatible with advanced manufacturing techniques such as additive manufacturing, they enable the localized production of sustainable goods, fostering community resilience and reducing reliance on global supply chains for raw materials. This decentralized production model can lead to the creation of ‘green’ jobs within the local recycling and manufacturing sectors, while simultaneously promoting public acceptance of waste-derived materials in high-value, functional applications [94,95].

The integration of sewage sludge-derived biochar into a recycled PET matrix transcends mere material enhancement: it addresses two critical waste management streams simultaneously. By repurposing municipal waste (sludge) to extend the life-cycle of post-consumer plastic (rPET), this research provides a tangible pathway for the circular economy. Table 5 outlines the multi-dimensional environmental and societal benefits of this waste-to-resource approach, highlighting its potential to reduce carbon footprints and mitigate microplastic pollution through enhanced UV stability.

## 5. Future Research for Impact Validation

While the experimental results presented in this study confirm the chemical integrity and thermal stability of the rPET/BC nanocomposites, a comprehensive validation of their sustainability profile is essential for industrial adoption. To approve the environmental safety and economic viability of these materials, a standardized research framework must be implemented. Table 6 proposes a strategic roadmap for future research, focusing on life-cycle impacts, toxicity safety, and the optimization of the material for advanced manufacturing techniques like additive manufacturing.

## 6. Conclusions

The present study demonstrates the successful incorporation of sewage sludge-derived BC into a recycled PET matrix for the development of sustainable nanocomposite materials with enhanced durability. Structural characterization confirms the absence of chemical interactions between the filler and the polymer matrix, while SEM observations reveal a homogeneous dispersion of BC particles in all filler loadings. DSC, XRD, and PLM analyses show that BC acts as a heterogeneous nucleating agent, accelerating crystallization, while TGA and Py–GC/MS results indicate a slight improvement in thermal stability, accompanied by reduced formation of volatile degradation products and increased char residue. The most significant finding of this work concerns the UV-aging performance of the materials. IV measurements reveal that the BC-containing nanocomposites experienced a smaller reduction in molecular weight after UV exposure compared to neat rPET, indicating that BC effectively mitigates photo-induced chain scission and contributes to the preservation of the polymer structure during aging. Overall, although the observed changes in the bulk properties are moderate due to the relatively low BC loadings, the consistency among the complementary characterization techniques demonstrates that sewage sludge-derived BC can be considered as a promising multifunctional additive for recycled PET. It should be emphasized that the conclusions of the present study are specific to the sewage sludge-derived BC investigated herein. Since the properties of BC strongly depend on its feedstock, particle size and morphology, pyrolysis conditions, ash content, and surface chemistry, biochars produced from different precursors or under different processing conditions may exhibit different behavior when incorporated into polymer matrices. The proposed approach enables the simultaneous valorization of two waste streams and supports the development of sustainable polymeric materials within a circular economy framework.

## 7. Future Perspectives

The results of the present study demonstrate the potential of sewage sludge-derived BC as a multifunctional stabilizing additive for recycled PET. Nevertheless, additional research is required to fully evaluate the long-term performance and environmental implications of these materials. Future studies should investigate the mechanical behavior of the nanocomposites before and after prolonged environmental aging, as well as their resistance to combined weathering conditions involving UV radiation, temperature fluctuations, and humidity. Isothermal crystallization experiments could provide a more comprehensive understanding of the crystallization kinetics and further elucidate the nucleating role of BC. In addition, particular attention should be given to the relationship between molecular weight retention, fragmentation resistance, and long-term durability. Furthermore, life-cycle assessment (LCA) and leaching studies are necessary to evaluate the overall environmental benefits and safety of sewage sludge-derived BC in polymer applications. Finally, the mechanisms responsible for the stabilizing effect of BC should be further explored through advanced characterization techniques, aiming to optimize filler characteristics and maximize the resistance of recycled polymers to thermal and photo-oxidative degradation.

## Figures and Tables

**Table 1:** Composition of rPET/BC nanocomposites prepared with varying BC loadings. Names reflect the respective BC concentrations.

Abbreviation	Composition (g)		
	rPET	BC	PMDA
**rPET**	49.75	-	0.25
**rPET/BC0.5**	49.50	0.25	0.25
**rPET/BC1**	49.25	0.50	0.25
**rPET/BC2.5**	48.50	1.25	0.25
**rPET/BC5**	47.25	2.50	0.25

**Table 2:** DSC, XRD, and TGA results of neat rPET and its biochar-filled nanocomposites.

Abbreviation	T_m_ (°C)	ΔH_m_ (J/g)	T_c_ (°C)	ΔH_c_ (J/g)	X_c_ (DSC)	X_c_ (XRD)	T_0.5%_ (°C)	T_max_ (°C)
**rPET**	244	32	187	37	23%	38%	396	474
**rPET/BC0.5**	245	30	190	36	22%	37%	387	476
**rPET/BC1**	246	32	196	41	23%	37%	389	476
**rPET/BC2.5**	244	29	199	40	21%	36%	387	477
**rPET/BC5**	245	28	199	38	21%	34%	386	478

**Table 3:** Characteristic thermal degradation products for rPET and rPET/BC5, along with the assigned compounds as interpreted by MS spectra.

Peak Annotation	Retention Time (min.)	Relative Peak Abundance (a.u.)		Assigned Compound
		rPET	rPET/BC5	
LB	0.93	98.8	n.d.	CO_2_
AC	1.59	52.2	100	Acetaldehyde
B	2.49	6.4	14.0	Benzene
A	8.96	9.7	n.d.	Vinyl benzoate
BA	11.06	51.7	65.4	Benzoic acid
BF	12.53	16.4	17.9	2-Butylbenzofuran
DP	12.80	19.8	14.0	Diphenyl
C	14.86	18.0	12.4	Divinyl terephthalate
D	16.49	6.4	7.5	4-(Vinyloxycarbonyl) benzoic acid
PB	18.22	7.3	6.8	Biphenyl-4-carboxylic acid
E	18.64	9.6	7.4	Ethan-1,2-diyldibenzoate
F	21.17	27.3	22.8	2-(Benzoyloxy)ethyl vinyl terephthalate
G	23.41	7.4	6.1	2-(4-((2-(Benzoyloxy)ethoxy)carbonyl)benzoyloxy)ethyl vinyl terephthalate
HB	24.95	41.9	24.8	2-(2-Hydroxyethoxy)ethyl benzoate
PE1	28.13	7.9	10.5	2-(Benzoyloxy)ethyl biphenyl-4-carboxylate
PE2	28.60	10.0	13.4	Ethan-1,2-diyl divinyl diterephthalate

**Table 4:** DSC results of quenched rPET and its biochar-filled nanocomposites before and after 90 days of UV irradiation.

Abbreviation	[η] (dL/g)	T_g_ (°C)	T_cc_ (°C)	ΔH_cc_ (J/g)	T_m_ (°C)	ΔH_m_ (J/g)
**rPET** (**0 Days**)	0.49 ± 0.01	78	136	40	246	45
**rPET** (**90 Days**)	0.38 ± 0.01	73	128	42	246 + 194	49 + 4
**rPET/BC0.5** (**0 Days**)	0.51 ± 0.01	79	136	41	246	48
**rPET/BC0.5** (**90 Days**)	0.42 ± 0.02	72	131	38	243	46
**rPET/BC1** (**0 Days**)	0.55 ± 0.01	78	133	36	247	44
**rPET/BC1** (**90 Days**)	0.48 ± 0.01	76	133	44	245	45
**rPET/BC2.5** (**0 Days**)	0.49 ± 0.01	74	135	44	245	45
**rPET/BC2.5** (**90 Days**)	0.43 ± 0.01	76	132	42	246	51
**rPET/BC5** (**0 Days**)	0.47 ± 0.01	78	138	44	244	48
**rPET/BC5** (**90 Days**)	0.44 ± 0.02	78	136	43	246	48

**Table 5:** Environmental and societal impacts of rPET/BC nanoomposites.

Aspect	Impact Description	Mechanism and Benefits
Environmental	Waste Valorization	Diverts sewage sludge from landfills/incineration and utilizes post-consumer rPET, promoting a “double-circular” economy [86].
Environmental	Carbon Sequestration	Converts organic waste into stable biochar, effectively locking carbon into a solid polymer matrix and reducing CO_2_ emissions.
Environmental	Microplastic Mitigation	Biochar acts as a UV stabilizer, slowing polymer photodegradation and reducing the fragmentation of products into microplastics.
Societal	Circular Economy Transition	Provides a value-added application for municipal waste, reducing management costs for local governments.
Societal	Sustainable Manufacturing	Supports localized production via additive manufacturing, reducing reliance on global raw material supply chains.
Societal	Green Job Creation	Encourages the growth of local industries centered around waste-to-resource technologies and advanced recycling.

**Table 6:** Proposed research framework for impact validation.

Research	Objective	Key Metrics and Methods
Life-Cycle Assessment (LCA)	Quantify the “cradle-to-grave” environmental footprint.	Global warming potential (GWP), cumulative energy demand (CED), and comparison with virgin PET.
Life-Cycle Costs (LCC)	Evaluate economic feasibility.	Cost–benefit analysis of sludge pyrolysis vs. commercial stabilizers and landfill fees.
Leaching and Toxicity	Ensure the material is safe for human and environmental contact.	Heavy metal migration tests (Pb, Cd, Cr, Ni) using standard leaching protocols (e.g., EN 12457-2).
Technoeconomic Analysis (TEA)	Evaluate the commercial and industrial feasibility.	Comparison of sludge pyrolysis costs vs. industrial UV stabilizer market prices and landfill fees.
Social LCA (S-LCA)	Assess societal impacts.	Evaluation of worker safety, public perception, and regional economic benefits.
Weathering and Durability	Validate the effectiveness of biochar as a long-term stabilizer.	Accelerated UV aging tests followed by mechanical testing and mass loss analysis for microplastic release.
Processability Study	Optimize the material for advanced manufacturing.	Rheological characterization to determine printability limits for additive manufacturing.

## Data Availability

The original contributions presented in this study are included in the article. Further inquiries can be directed to the corresponding author.

## Supplementary Materials

The following supporting information can be downloaded at: https://www.mdpi.com/article/10.3390/polym18141758/s1.
