# Supplementary material for: Utilization of Sewage Sludge-Derived Biochar as a Functional UV Stabilizer in Recycled Poly(ethylene terephthalate) Nanocomposite Materials
Source: Polymers (Basel). 2026 Jul 19;18(14):1758. doi: 10.3390/polym18141758 (PMC13417677; doi:10.3390/polym18141758)
Supplement: Supplementary file 1 [file polymers-18-01758-s001.zip › polymers-4413089-supplementary.pdf]

polymers

MDPI

Article

Utilization of Sewage Sludge-Derived Biochar as a Functional UV Stabilizer in Recycled Poly(ethylene terephthalate) Nanocomposite Materials

Nikolaos Pardalis [1], Lazaros Karagiannidis [2], Panagiotis A. Klonos [3], Eleftheria Maria Pechlivani [4,*], Rafail O. Ioannidis [1], Apostolos Kyritsis [3], Konstantinos Chrisafis [2] and Dimitrios N. Bikiaris [1]

1 Laboratory of Polymer Chemistry and Technology, Department of Chemistry, Aristotle University of Thessaloniki, GR54124 Thessaloniki, Greece; npardal@chem.auth.gr (N.P.); rafailio@chem.auth.gr (R.O.I.); dbic@chem.auth.gr (D.N.B.)
2 Laboratory of Advanced Materials and Devices, Department of Physics, Aristotle University of Thessaloniki, GR54124 Thessaloniki, Greece; lkaragi@physics.auth.gr (L.K.); hrisafis@physics.auth.gr (K.C.)
3 Dielectrics Research Group, Department of Physics, National Technical University of Athens, GR15780 Athens, Greece; pklonos@central.ntua.gr (P.A.K.); akyrits@central.ntua.gr (A.K.)
4 Centre for Research and Technology Hellas, Information Technologies Institute, 6th km Charilaou-Thermi Road, GR57001 Thessaloniki, Greece
* Correspondence: riapechl@iti.gr

Supplementary Information

The IR spectrum of sewage sludge-derived BC (Figure S1) reveals several characteristic bands. A strong peak at 1049 cm^{-1}, assigned to P–O stretching vibrations of phosphate groups, dominates the spectrum, while another intense band at 578 cm^{-1} corresponds to metal–oxygen (M–O) vibrations, such as Fe–O or Ca–O, reflecting the mineral-rich nature of the sludge feedstock [26]. Additional features include shoulders at ~914 and 975 cm^{-1}, attributed to Si–O stretching from silicate or aluminosilicate phases. Weaker bands are also observed in the 1650–1750 cm^{-1} region, corresponding to C=O stretching, and at ~1514 cm^{-1}, linked to aromatic C=C vibrations, confirming the partial aromatic character of the char matrix [20]. A broad band between 3600–3800 cm^{-1} is assigned to hydroxyl groups (–OH) from phenolic, alcoholic, or adsorbed water functionalities [22]. Considering the relatively low BC loading in the present study (0.5–5% wt.), the characteristic absorption bands of BC are expected to be masked by the stronger and well-defined absorption bands of the rPET matrix. In addition, several BC bands are inherently broad and overlap with characteristic PET absorptions, further limiting their detectability in the nanocomposite spectra. Consequently, the absence of distinct BC-related peaks in the IR spectra of the nanocomposites should not be interpreted as the absence of the filler, but rather as a consequence of its low concentration and the spectral dominance of the polymer matrix.

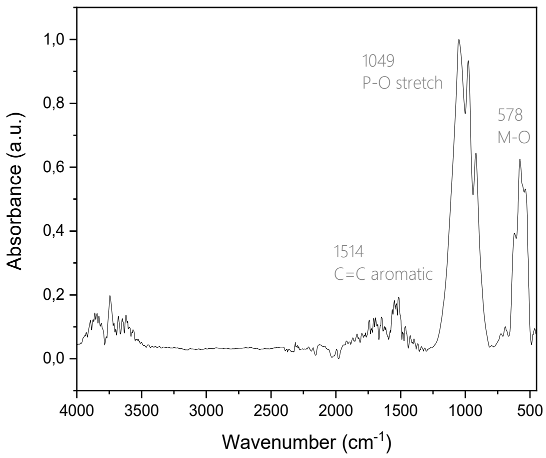

Figure S1. IR spectrum of sewage sludge-derived biochar.

SEM analysis (Figure S2) shows that the sewage sludge-derived BC is composed of irregularly shaped particles with rough surface morphology. The predominance of submicron particles is consistent with the average particle size determined by DLS. A small number of larger agglomerates are also visible, which most likely arise from the natural aggregation of the BC powder during sample preparation.

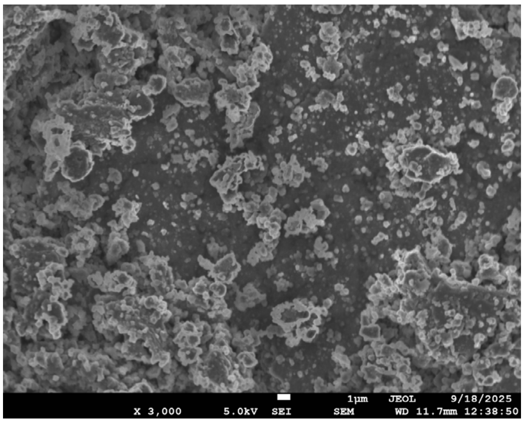

Figure S2. SEM observation of biochar. IR spectra of neat rPET and rPET/BC nanocomposites before and after 90 days of accelerated UV aging.

The XRD pattern of the sewage sludge-derived biochar (Figure S3) exhibits a broad amorphous halo between 20 and 35° (2θ), characteristic of disordered carbon structures. Superimposed sharp diffraction peaks indicate the presence of crystalline inorganic mineral phases originating from the sewage sludge feedstock, such as silicates, phosphates, and metal-containing compounds.

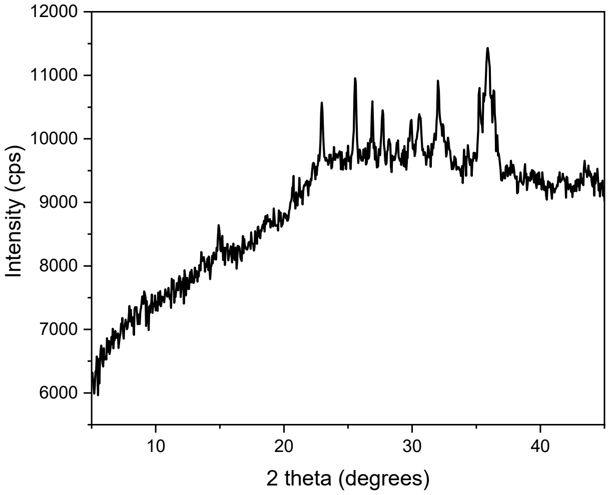

Figure S3. XRD pattern of sewage sludge-derived biochar.

The IR spectra of the UV-aged samples remained essentially unchanged after irradiation, with no detectable formation of new absorption bands or significant changes in the characteristic bands of rPET (Figure S4). This behavior suggests that photo-oxidation under these specific conditions was insufficient to produce detectable changes in the FTIR spectra. Since PET already exhibits a strong ester carbonyl band, the formation of relatively small amounts of oxidation products is expected to be masked by the dominant absorptions of the polymer. Consequently, ATR-FTIR appears less sensitive than IV for detecting the early stages of photo-induced degradation.

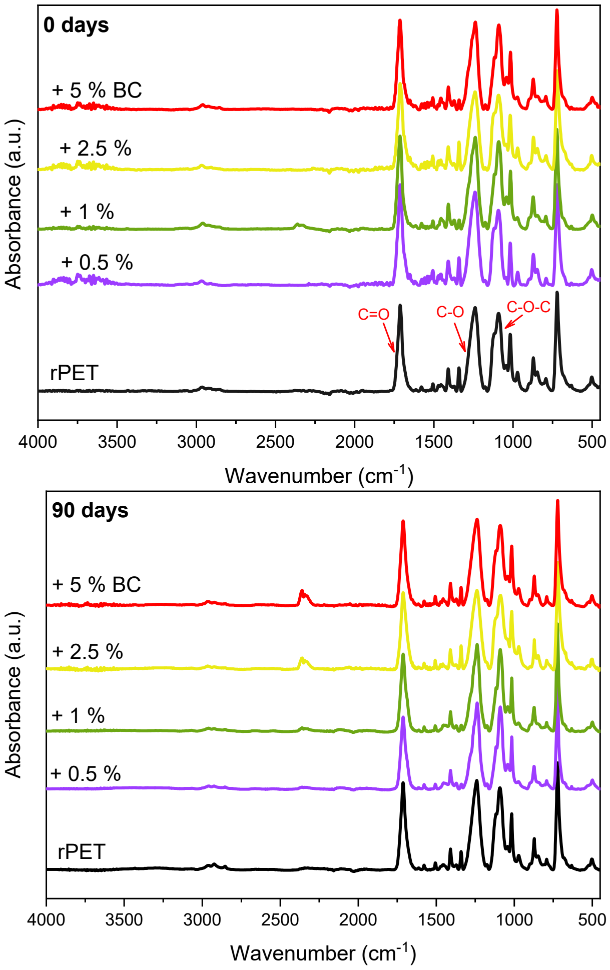

Figure S4. IR spectra of neat rPET and rPET/BC nanocomposites before and after 90 days of accelerated UV aging.

References

20. Bezabeh, M.W.; Krogstad, T.; Eich-greatorex, S. Characterisation of Biochar from Manure and Sewage Sludge Using XRD and FTIR. **2025**.

22. Jin, J.; Li, Y.; Zhang, J.; Wu, S.; Cao, Y.; Liang, P.; Zhang, J.; Wong, M.H.; Wang, M.; Shan, S.; et al. Influence of Pyrolysis Temperature on Properties and Environmental Safety of Heavy Metals in Biochars Derived from Municipal Sewage Sludge. *Journal of Hazardous Materials* **2016**, *320*, 417–426, doi:10.1016/j.jhazmat.2016.08.050.

26. Fan, X.; Qian, Z.; Liu, J.; Geng, N.; Hou, J.; Li, D. Investigation on the Adsorption of Antibiotics from Water by Metal Loaded Sewage Sludge Biochar. *Water Science and Technology* **2021**, *83*, 739–750, doi:10.2166/wst.2020.578.

Disclaimer/Publisher's Note: The statements, opinions and data contained in all publications are solely those of the individual author(s) and contributor(s) and not of MDPI and/or the editor(s). MDPI and/or the editor(s) disclaim responsibility for any injury to people or property resulting from any ideas, methods, instructions or products referred to in the content.
